# Clinical and CT/MRI features of hepatic AL amyloidosis: preliminary experience in 10 cases

**DOI:** 10.3389/fonc.2026.1675220

**Published:** 2026-06-19

**Authors:** Yanyan Zhang, Wei Wang, Wenyan Song, Jing Chang, Hongjun Li

**Affiliations:** 1 Department of Diagnostic Radiology, Beijing You’an Hospital, Capital Medical University, Beijing, China; 2 Department of Pathology, Beijing You’an Hospital, Capital Medical University, Beijing, China

**Keywords:** computed tomography, delayed enhancement, hepatic AL amyloidosis, hepatomegaly, magnetic resonance imaging

## Abstract

**Purpose:**

To identify the key clinical and imaging clues on computed tomography (CT) and magnetic resonance imaging (MRI) that can suggest the diagnosis of hepatic AL amyloidosis, thereby improving diagnostic accuracy and timely recognition among peers.

**Methods:**

We retrospectively analyzed the clinical, CT, and MRI findings of 10 patients with confirmed hepatic AL amyloidosis.

**Results:**

The predominant clinical manifestations included hepatomegaly (10/10, 100%), abdominal distension (9/10, 90%), fatigue (4/10, 40%), and weight loss (3/10, 30%). Laboratory findings showed markedly elevated alkaline phosphatase (ALP) and gamma-glutamyl transferase (GGT) (10/10, 100%), with normal or mildly elevated transaminases; hypoalbuminemia was present in all patients (10/10, 100%). CT imaging demonstrated diffuse hepatomegaly with decreased hepatic attenuation in all 8 patients who underwent CT. MRI (6 patients) showed marked hepatomegaly with smooth margins, mildly and uniformly increased T2WI signal, along with vascular rarefaction. Contrast-enhanced CT/MRI revealed delayed enhancement. Additionally, poor visualization or stenosis of the main hepatic veins and periportal edema were also observed in all cases (10/10, 100%). MRCP was performed in 5 patients, and none showed evidence of biliary obstruction. Splenic hypoperfusion was observed in 7 patients, and renal hypoperfusion in 2 patients.

**Conclusions:**

Despite its low incidence, hepatic AL amyloidosis should be suspected in patients presenting with unexplained hepatomegaly, markedly elevated ALP/GGT with relatively preserved transaminases, and specific imaging findings such as delayed enhancement, poor visualization or stenosis of the main hepatic veins, and periportal edema. Recognition of these clues, along with extrahepatic findings such as splenic or renal hypoperfusion, can significantly improve diagnostic accuracy and timely recognition of this disease.

## Introduction

Amyloidosis is a rare progressive disease characterized by the extracellular deposition of pathologic insoluble fibrillar proteins in various organs and tissues (1), and the accumulation of amyloid proteins disrupts tissue structure, ultimately leading to organ dysfunction and failure. According to the latest statistics from the National Institute of Health (NIH), the incidence of AL (amyloid light chain) amyloidosis in Western countries is approximately 1 case per 100,000 person-years (2). In amyloidosis, the organs most frequently involved are as follows: heart (70-80%) (3, 4), kidneys (60-70%) (5), liver, gastrointestinal tract, nervous system, respiratory tract, breast, lungs, and musculoskeletal tissues. Although the liver is a common site of amyloid deposition, hepatic involvement rarely presents with specific clinical manifestations and often manifests as nonspecific symptoms, with only 0.7% of patients presenting with hepatic involvement as the initial manifestation (6), resulting in a diagnostic delay of more than one year (5, 7). In fact, only 26% of patients had amyloidosis considered in their initial differential diagnosis (8, 9). Although rare, hepatic amyloidosis carries a poor prognosis; therefore, a high index of suspicion is crucial for early diagnosis. We conducted a retrospective analysis of imaging characteristics and clinical manifestations in 10 cases of hepatic AL amyloidosis, aiming to improve diagnostic accuracy and patient outcomes for this rare disorder.

## Materials and methods

### Patients’ information

This retrospective analysis was approved by our institutional review board, and the requirement for patient consent was waived. We identified 10 patients with confirmed hepatic AL amyloidosis who were treated at our institution between January 2013 and March 2025. The clinical data (including demographic characteristics, laboratory results and clinical symptoms) and pathology reports were obtained from the electronic medical records. Meanwhile, we identified the laboratory results with the shortest temporal interval to the imaging examination.

### CT and MRI techniques

Eight patients underwent Computed tomographic (CT) examinations, using a 64-detector row scanner system (LightSpeed VCT 64, GE Healthcare, Waukesha, Wisconsin, USA). The scanning parameters were as follows: 120 kV voltage, 200 mA current, 5 mm section thickness. Using Iopromide (Ultravist 370, Bayer Schering Pharma, Berlin, Germany) as the CT contrast agent, with a dose: 1.5 mL/kg, and injection flow rate: 3 mL/s. Finally, a 20 mL saline flush was injected at a rate of 3 mL/s. Serial dynamic contrast-enhanced scans were obtained on during the hepatic arterial phase (AP) (25–40 s), portal venous phase (PVP) (60–75 s) and delayed phase (DP) (100–120 s) after the contrast injection.

Six patients underwent magnetic resonance imaging (MRI), two of whom underwent DCE-MRI. MRI examination was performed on a Magnetom Verio 3.0T (Trio, Siemens Healthineers, Erlangen, Germany) and an Ingenia 3.0T (GE, SIGNA Pioneer) with an 8-channel phased array body coil. The conventional MRI protocol included unenhanced axial T1-weighted imaging (T1WI); axial and coronal T2-weighted imaging (T2WI); and axial, coronal, and sagittal contrast-enhanced T1WI. Diffusion-weighted single-shot echo-planar imaging (DWI) with b values of 0 and 800 s/mm^2^ was performed before contrast agent administration. The parameters of the T1-weighted fast low angle shot sequence were mentioned as below: TR/TE, 170/2.30; out-of-phase TE, 3.67 ms; matrix size, 256 × 205; flip angle, 65°. The three-dimensional volumetric interpolated breath-hold examination (3D-VIBE) sequence was obtained before (pre-contrast) and after the injection of contrast agent (Gd-BOPTA, MultiHance, Bracco Pharma, Italy) at a rate of 2 mL/s. The serial dynamic contrast-enhanced scans including AP, PVP and EP were collected at the following times: 25–40 s, 45–90 s and 2–5 min.

### Image analysis

The imaging findings were systematically evaluated according to the following parameters: 1) Hepatomegaly, characterized by abnormal, diffuse hepatic enlargement, the liver exhibits an enlarged craniocaudal diameter of ≥ 15.5 cm along the midclavicular line (10); 2) Liver parenchyma attenuation/intensity (hypo-, iso-, or hyper-attenuation on CT; corresponding signal intensity on MRI); 3) Ascites (present/absent); 4) Enhancement pattern(homogeneous/heterogeneous enhancement; early/delayed enhancement); 5) Periportal edema, manifested as: I Hypoattenuating halos or linear structures surrounding portal vessels on CT; II Hyperintense linear structures on T2-weighted MR imaging (11); 6) Splenic hypoperfusion, characterized by the lack of parenchymal enhancement and T2-weighted MR imaging showed decreased signal intensity (12, 13); 7). Hepatic venous abnormality (stenosis/occlusion), poor or non-visualization of main hepatic veins, or presence of thrombus within them. Based on these findings, image interpretation was performed retrospectively and blindly by two experienced reviewers (20 and 10 years of experience, respectively) in consensus.

### Pathology and amyloid typing

**Figure 1:**
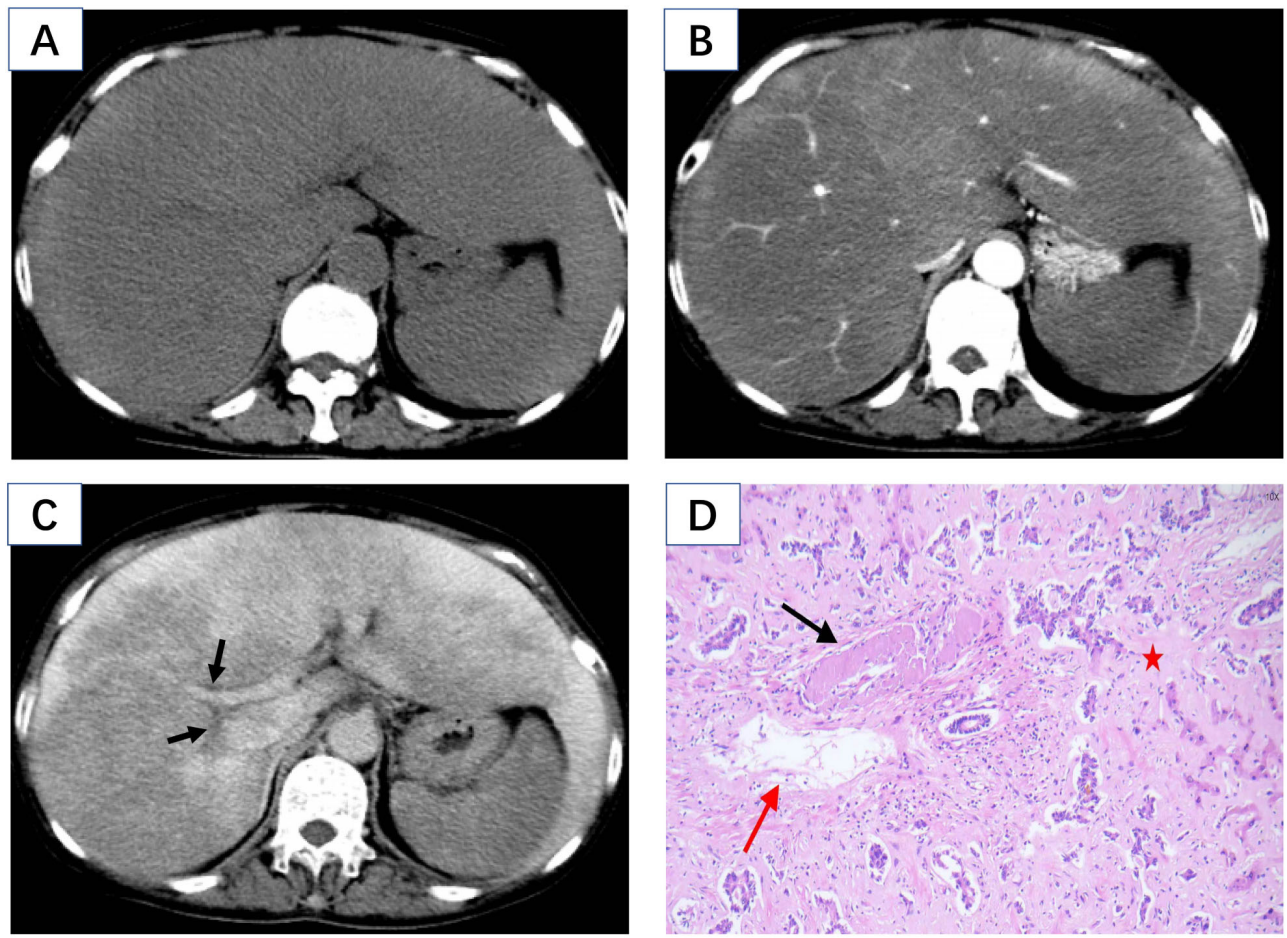
A 63-year-old female diagnosed with hepatic AL amyloidosis. **(A)** Non-contrast CT axial shows hepatomegaly with diffuse parenchymal hypodensity; **(B)** Dynamic contrast-enhanced CT demonstrates a mild mottled enhancement pattern in the arterial phase; **(C)** Delayed phase exhibits progressive parenchyma enhancement with periportal edema (arrow); **(D)** Histopathology (HE staining, ×200) revealed extensive deposits of homogeneous, amorphous eosinophilic material obstructing hepatic sinusoids (red star), arterial lumens are fully occluded by amyloid deposits (black arrow), while venous lumens remained unaffected (red arrow).

Histological examination revealed extensive deposits of homogeneous, amorphous material, within the hepatic sinusoids and central veins, which appeared pink under normal light when stained with Congo red (**Figure 1D**). Amyloid deposits were also observed within the arterial walls and portal vein. The diagnosis was confirmed by consensus of two senior pathologists. Congo red staining was positive in all cases. Among them, the diagnosis of AL amyloidosis was confirmed in 2 cases by immunohistochemistry, in 7 cases by serum/urine immunofixation electrophoresis combined with free light chain assay, and the remaining 1 case was identified as AL amyloidosis associated with plasma cell neoplasm.

## Results

### Baseline clinical and laboratory characteristics

Among the 10 patients with hepatic AL amyloidosis (7 males and 3 females), the mean age was 57.3 years (range: 46–73 years). The primary presenting symptoms included abdominal distension (9/10, 90%), fatigue (4/10, 40%), and weight loss (3/10, 30%); anorexia and jaundice were also noted. Liver function test showed that AST and ALT were normal or only slightly elevated. In contrast, the cholestasis markers ALP and GGT were markedly elevated in all patients (100%). Specifically, GGT levels were at least 2-fold above the upper limit of normal (ULN), reaching up to over 40-fold (maximum 43-fold), while ALP levels exceeded 1.5-fold ULN, with a maximum of up to 7-fold. Additionally, albumin (ALB) levels, reflecting liver synthetic function, were decreased in all cases (100%), indicating hypoalbuminemia in every patient. Prothrombin time (PT) was prolonged in 80% (8/10) of the patients, and hyperbilirubinemia developed in 70% (7/10). The clinical characteristics and laboratory results are presented in **Table 1**.

**Table 1:** Clinical findings and laboratory results in 10 patients with hepatic AL amyloidosis.

No	Age(y)	sex	Extrahepatic involvement	Abdominal distension	fatigue	Weight loss	ALT (U/L)	AST(U/L)	TBIL (μmol/L)	DBIL(μmol/L)	TBA (μmol/L)	ALP(U/L)	GGT (U/L)	ALB (g/L)	PT(s)
1	60	M	-	+	-	-	15.1	40	10.8	4.3	38.5	458.9	2601.3	29	11.2
2	63	F	-	+	-	-	19	45	28.4	19.8	104.3	197	125	33.6	12.9
3	52	M	Heart	+	+	+	74	106	27.6	26.2	39.6	879	759	26.7	15.0
4	53	M	-	+	+	+	44	87	67.5	51.3	112.6	624	1197	33.5	15.6
5	54	M	Heart	+	+	-	28	75	101.7	85.1	54.9	759	295.4	24.6	14.9
6	65	M	Kidney, Heart	+	-	-	169	282	58.9	44.3	213.7	859	530	29.4	15.3
7	73	M	Kidney	+	-	-	15	56	85.9	62.4	33.4	539	425	27.8	24.5
8	58	F	Kidney	+	+	+	16	35	19.7	6.5	10.3	413	716	37.3	11.7
9	46	M	Kidney	-	-	-	24	30	223.8	181.8	169.8	616	262	26.3	13.2
10	49	F	-	+	-	-	17.9	46.4	19.4	9.7	18.5	336	330	25.3	15.8

+ yes/present/positive, - no/absent/negative; ALT(9–50 U/L); AST(15–40 U/L); TBIL(5-21μmol/L); DBIL(<7μmol/L); TBA(<10μmol/L); ALP(45–125 U/L); ALB (40–55 g/L); GGT(10–60 U/L); PT (9.9-12.8 s).

### CT and MRI findings

**Figure 2:**
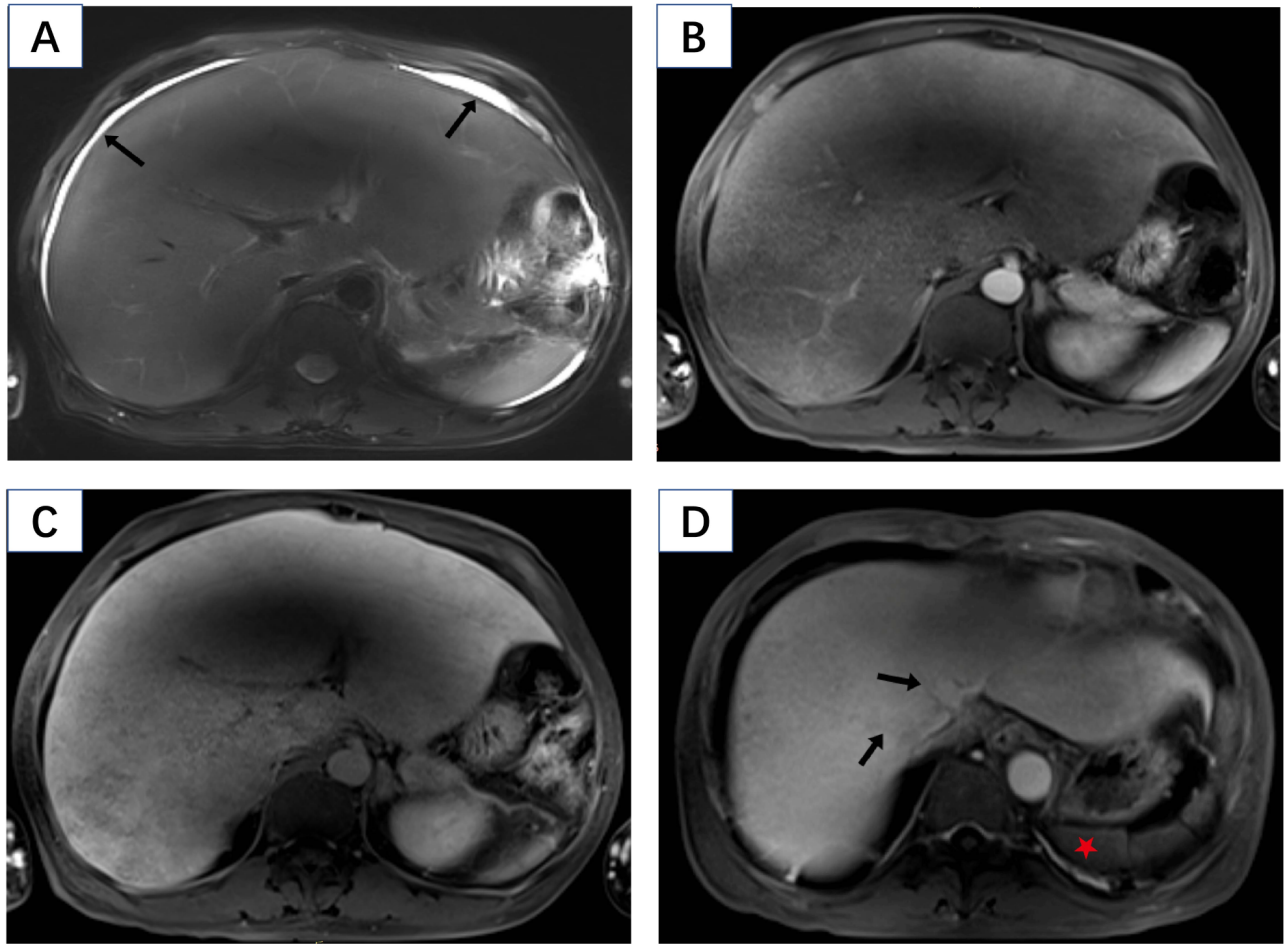
Hepatic AL amyloidosis in a 60-year-old male **(A–C)**. (A) Axial T2-weighted MRI demonstrates hepatomegaly with finely textured hepatic parenchyma and perihepatic ascites (arrow). **(B)** Arterial phase of DCE-MRI shows mild enhancement of the hepatic parenchyma. **(C)** Equilibrium phase of DCE-MRI reveals delayed enhancement of the hepatic parenchyma. **(D)** Equilibrium phase of DCE-MRI in another 52-year-old male patient displays delayed parenchymal enhancement, with hepatic venous stenosis (arrow) and splenic hypoperfusion (red star).

The imaging features of hepatic amyloidosis are summarized in **Table 2**. All imaging data were retrospectively and blindly analyzed by two experienced radiologists (with 20 and 10 years of experience, respectively). The most significant imaging manifestations included hepatomegaly (100%), periportal edema (100%), and poor visualization or stenosis of the main hepatic veins (100%). Hepatomegaly is the most prominent imaging feature, with a marked increase in liver volume. On non-contrast CT, the liver parenchyma demonstrates diffuse low attenuation (**Figure 1A**), requiring differentiation from hepatic steatosis. On MRI, the liver exhibits mildly and uniformly increased T2-weighted imaging (T2WI) signal, along with vascular rarefaction (**Figure 2A**). The absence of signal loss on T1-weighted in-phase/opposed-phase (chemical shift) imaging excluded significant fatty deposition. On contrast-enhanced imaging, all patients exhibited mild patchy peripheral enhancement in the arterial phase (**Figures 1B**, **2B**), with delayed progressive parenchymal enhancement in the portal and delayed phases (**Figure 2C**). Periportal edema manifested as periportal tracking (**Figure 1C**). The main hepatic veins were abnormal in all cases, showing either poorly visualization or stenosis (**Figure 2D**). Furthermore, MRCP revealed no obvious dilatation or stenosis of the intrahepatic or extrahepatic bile ducts, excluding obstructive etiologies of hyperbilirubinemia.

**Table 2:** The imaging findings in 10 patients with hepatic AL amyloidosis.

No	Imaging protocol	Hepatomegaly	Ascites	Delayed enhancement	Periportal edema	Homogeneous parenchyma	Splenic hypoperfusion	Kidney hypoperfusion	Hepatic venous abnormality
1	DCE-MRI/MRCP	+	+	+	+	+	-	-	+
2	CE-CT	+	-	+	+	+	+	-	+
3	DCE-MRI	+	+	+	+	+	+	+	+
4	CE-CT	+	+	+	+	+	+	-	+
5	CE-CT/MRCP	+	+	+	+	+	+	-	+
6	CE-CT/MRCP	+	+	+	+	+	-	-	+
7	CE-CT/MRCP	+	+	+	+	+	-	+	+
8	CE-CT	+	+	+	+	+	+	-	+
9	CE-CT/MRCP	+	+	+	+	+	+	-	+
10	CE-CT	+	+	+	+	+	+	-	+

+ yes/present/positive, - no/absent/negative; CE-CT: Contrast-enhanced CT; DCE-MRI: Dynamic contrast-enhanced MRI.

Splenomegaly is uncommon in amyloidosis, but spleen hypoperfusion (**Figure 2D**) occurred in 70% (7/10) of the 10 patients. Ascites and renal hypoperfusion may also be present, with incidence rates of 90% (9/10) and 20% (2/10), respectively.

## Discussion

The liver plays a unique role in various types of amyloidosis, serving both as a target organ for protein misfolding and as a source of it (14, 15). Among the 42 soluble amyloid proteins identified so far (4, 16), the types most frequently affecting the liver are AL, ATTR, AA, and amyloid leukocyte chemotactic factor-2 (ALECT2) (17). AL is the most common form of hepatic amyloidosis; ALECT2 amyloidosis is the second most common cause of hepatic amyloidosis in the United States (18). Meanwhile, the liver is the second most commonly affected organ in AA amyloidosis after the kidneys, accounting for approximately 20% of cases (15). The prevalence of liver disease in patients with ATTR is high (41.3%), which is mainly associated with hepatic congestion resulting from cardiac dysfunction (19).

The incidence of amyloidosis rises with age, and hepatic amyloidosis predominantly affects elderly males, with a mean age of 63 years (20), with nearly 90% of patients over the age of fifty. Our cohort demonstrated a similar age distribution pattern (80% > 50 years) but with a relatively younger median age (56 years). Patients present with nonspecific clinical manifestations, including abdominal distension, fatigue, and liver function abnormalities. Jaundice and weight loss may also be observed. Hepatomegaly that is disproportionate to the degree of hepatocellular dysfunction, along with significantly elevated levels of ALP and GGT, represents the most specific indicators of hepatic amyloidosis. The data from our group all showed hepatomegaly with significantly elevated ALP and GGT levels. ALP levels exceeded 1.5 times the ULN, and GGT levels exceeded 2 times the ULN. Notably, some patients exhibited extremely high levels, with ALP up to 879 U/L and GGT up to 2601.3 U/L.

Early diagnosis of hepatic amyloidosis remains a major challenge. On unenhanced CT, in addition to hepatomegaly, we also observed decreased liver attenuation, whereas on MRI, no fat component was detected on opposed-phase imaging, which may be attributed to amyloid deposition. Mild mottled enhancement in the arterial phase, followed by delayed enhancement in the portal venous and delayed phases, was a characteristic feature shared by all cases in this group and has also been reported previously (13, 21, 22). We believe that these unique features may reflect reduced perfusion caused by heterogeneous deposition of amyloid proteins in the perisinusoidal space and vessel walls, with arterial involvement being more prominent, ultimately leading to a prolonged time for the liver to reach peak intensity. Wang et al. described this enhancement as “Linghua – window” patterns (23). These findings are distinctly different from the “cloud-like” heterogeneous enhancement seen in hepatic sinusoidal obstruction syndrome (HSOS) and the “geographic” enhancement patterns of Budd-Chiari syndrome (BCS). Additionally, all patients demonstrated poor visualization or stenosis of the main hepatic veins on imaging, as well as periportal edema. A possible explanation is that the deposition of abundant amorphous amyloid material beneath the hepatic sinusoidal endothelial cells — specifically in the space of Disse — subsequently triggers a series of pathological alterations (1) hepatocyte atrophy, (2) progressive hepatic parenchymal expansion, (3) mechanical compression of hepatic veins, and consequent reduction in passive venous return and lymphatic drainage. However, this requires further validation. These imaging features can be radiologically indistinguishable from the hepatic venous insufficiency observed in HSOS (24), therefore, some scholars speculate that HSOS is a rare characteristic of liver involvement in systemic amyloidosis (25). Our study also found that a considerable number patients had accompanying splenic involvement, manifested as splenic hypoperfusion rather than splenomegaly. Renal involvement was also observed, with imaging findings showing renal hypoperfusion. These findings may be attributed to vascular damage and amyloid deposition in the spleen or kidneys. Such manifestations have received little attention in previous reports and may serve as a breakthrough point for the diagnosis of amyloidosis.

The clinical presentation and imaging features of hepatic AL amyloidosis demonstrate considerable overlap with HSOS, acute BCS, and acute hepatitis, particularly with HSOS. Radiologists need to integrate etiological factors, laboratory indicators, and subtle imaging signs for differential diagnosis (detailed in **Table 3**). Although the imaging findings of hepatic AL amyloidosis on CT and MRI are nonspecific, these features can still provide important clues for identifying early signs of the disease. Together with the patient’s laboratory indicators, they constitute the “red flags” for clinical suspicion of hepatic amyloidosis. It should be emphasized that the diagnostic gold standard remains liver biopsy; if clinically indicated, biopsy at an alternative site (such as the abdominal fat pad or kidney) may also be performed, followed by Congo red staining and necessary subtyping (including immunohistochemistry or mass spectrometry) to confirm the diagnosis. Hepatic amyloidosis has been described as a rapidly progressive disease associated with a poor prognosis (4), with a median survival of only 8.5 months (8), and an even poorer prognosis in patients with jaundice (4, 25, 26). Current evidence suggests that prognosis correlates directly with the extent of amyloid deposition (15). This strong association further underscores the critical importance of early diagnosis for timely therapeutic intervention.

**Table 3:** Differential diagnosis among hepatic amyloidosis, hepatic sinusoidal obstruction syndrome (HSOS), acute hepatitis, and acute Budd-Chiari syndrome (BCS).

Identification points	Hepatic amyloidosis	HSOS	Acute hepatitis	Acute BCS
Etiology	Amyloid deposition of unknown etiology	Clear history of pyrrolizidine alkaloid (PA) exposure; related to hematopoietic stem cell transplantation (HSCT); or associated with prior radiotherapy/chemotherapy	Viral, drug-induced, or alcoholic causes, etc.	Hepatic venous outflow obstruction at the level of the main hepatic veins or inferior vena cava due to various primary or secondary etiologies
Symptoms and signs	Abdominal distension, fatigue, jaundice and weight loss	Fatigue, abdominal distension, right upper quadrant pain, jaundice, ascites, and a rapidly enlarging tender liver	Fatigue, anorexia, jaundice (skin and scleral icterus), dark urine, itching, and right upper quadrant tenderness	Right upper quadrant pain, rapidly progressive hepatomegaly with tenderness, rapidly increasing ascites, accompanied by possible jaundice and decreased urine output
Laboratory findings	Markedly elevated ALP and GGT; normal or only mildly elevated ALT/AST; normal or only mildly abnormal ALB and PT	Markedly elevated TBIL, mildly to moderately elevated ALT and AST, decreased ALB, and prolonged PT	Markedly elevated ALT/AST; mild to moderate elevation of TBIL; normal ALB; normal or mildly prolonged PT	Markedly elevated ALT/AST, with normal or mildly abnormal ALB and PT
Imaging Findings	Hepatomegaly, “Linghua – window” patterns and delayed enhancement, poor visualization of hepatic veins; periportal edema; hypoperfusion of spleen or kidneys	Hepatomegaly, decreased liver density, “cloud-like” heterogeneous enhancement; Thin or indistinct hepatic veins with the “clover” sign; periportal edema	Normal or mildly enlarged liver size; gallbladder wall edema (“double-wall sign”); periportal edema	Diffuse marked hepatomegaly, geographic enhancement, thrombi in the hepatic veins and/or inferior vena cava, and massive ascites
Prognosis	Poor prognosis	Good prognosis	Good prognosis	Good prognosis

Several limitations of this study should be acknowledged. First, the relatively small sample size, while inevitable given the rarity of this condition, may restrict the generalizability of our results. Second, the imaging findings reported in this study are derived from a combination of CT and MRI examinations, each with different technical parameters and diagnostic performance. The heterogeneity of imaging modalities may have introduced bias. Third, our criteria for defining certain imaging features as clinically relevant disease markers were primarily qualitative, based on visual assessment and expert consensus rather than on quantitative perfusion parameters or standardized thresholds. Although this approach reflects real-world clinical practice, it inevitably introduces a degree of subjectivity and may be influenced by inter-observer variability. Future prospective multicenter studies with standardized protocols are needed to accumulate larger patient cohorts and to adopt unified imaging protocols with quantitative biomarkers (e.g., perfusion parameters). We believe that integrating precise subtyping, quantitative imaging, and collaborative data sharing will be key to advancing our understanding of hepatic amyloidosis and promoting clinical progress.

## Conclusions

Although hepatic AL amyloidosis is a rare clinical entity characterized by nonspecific clinical and imaging findings, it can still be considered in the differential diagnosis. Based on the findings of hepatomegaly and markedly elevated ALP and GGT levels, if radiologists also observe delayed hepatic parenchymal enhancement, poor visualization or stenosis of the main hepatic veins, and periportal edema, they should raise strong suspicion of hepatic AL amyloidosis. Such suspicion is the first step toward a definitive diagnosis.

## Data Availability

The original contributions presented in the study are included in the article/supplementary material. Further inquiries can be directed to the corresponding author.
